# Assessing the allelotypic effect of two aminocyclopropane carboxylic acid synthase-encoding genes *MdACS1* and *MdACS3a* on fruit ethylene production and softening in *Malus*

**DOI:** 10.1038/hortres.2016.24

**Published:** 2016-05-18

**Authors:** Laura Dougherty, Yuandi Zhu, Kenong Xu

**Affiliations:** 1 Horticulture Section, School of Integrative Plant Science, Cornell University, New York State Agricultural Experiment Station, Geneva, NY 14456, USA

## Abstract

Phytohormone ethylene largely determines apple fruit shelf life and storability. Previous studies demonstrated that *MdACS1* and *MdACS3a*, which encode 1-aminocyclopropane-1-carboxylic acid synthases (ACS), are crucial in apple fruit ethylene production. *MdACS1* is well-known to be intimately involved in the climacteric ethylene burst in fruit ripening, while *MdACS3a* has been regarded a main regulator for ethylene production transition from system 1 (during fruit development) to system 2 (during fruit ripening). However, *MdACS3a* was also shown to have limited roles in initiating the ripening process lately. To better assess their roles, fruit ethylene production and softening were evaluated at five time points during a 20-day post-harvest period in 97 *Malus* accessions and in 34 progeny from 2 controlled crosses. Allelotyping was accomplished using an existing marker (ACS1) for *MdACS1* and two markers (CAPS_866_ and CAPS_870_) developed here to specifically detect the two null alleles (*ACS3a-G289V* and *Mdacs3a*) of *MdACS3a*. In total, 952 *Malus* accessions were allelotyped with the three markers. The major findings included: The effect of *MdACS1* was significant on fruit ethylene production and softening while that of *MdACS3a* was less detectable; allele *MdACS1–2* was significantly associated with low ethylene and slow softening; under the same background of the *MdACS1* allelotypes, null allele *Mdacs3a* (not *ACS3a-G289V*) could confer a significant delay of ethylene peak; alleles *MdACS1–2* and *Mdacs3a* (excluding *ACS3a-G289V*) were highly enriched in *M. domestica* and *M. hybrid* when compared with those in *M. sieversii*. These findings are of practical implications in developing apples of low and delayed ethylene profiles by utilizing the beneficial alleles *MdACS1-2* and *Mdacs3a*.

## Introduction

To make fresh apple fruit available year-round for consumers, the controlled atmosphere (CA) storage technology has been adapted widely in the apple industry. The technology primarily employs low temperature, low O_2_ and high CO_2_ in combination with an ethylene production inhibitor 1-methylcyclopropene and others. Apple fruit can be stored for >10 months under optimal CA conditions. However, physiological disorders associated with CA storage, such as injuries induced by cold and CO_2_ and flesh browning induced by 1-methylcyclopropene, can cause substantial loss for storage operators.^1–3^ Such storage disorders have been reported for major apple varieties such as ‘Empire’ and ‘McIntosh’^2,4^ and for rising cultivars such as ‘Honeycrisp’.^3^ A strong need for new apples of long-shelf life and improved keeping quality with few or no storage disorders exists.

The gaseous phytohormone ethylene plays an important role in climacteric fruit ripening. The shelf life and storability of apple fruit are closely correlated with their ethylene production levels. Plant ethylene biosynthesis has been well-defined in Yang cycle that involves three enzymes: *S*-adenosylmethionine synthase, 1-aminocyclopropane-1-carboxylic acid (ACC) synthase (ACS) and ACC oxidase (ACO).^5^ The enzymes ACS and ACO have been the subject of extensive studies to better understand plant ethylene production. Studies in many plant species including tomato and apple have shown that ACS and ACO are encoded by gene families of multiple members, that is, the ACS family and the ACO family, respectively.

There are two systems of ethylene production in plants: system 1 occurs during plant/fruit growth and development; and system 2 is defined exclusively for the floral senescence and fruit-ripening stages.^6^ In tomato, system 1 ethylene biosynthesis involves LeACS6, 1A and LeACO1, 3, 4; whereas system 2 uses LeACS2, 4 and ACO1, 4.^7^ In apple, at least five *ACS* (*MdACS1–5*) and four *ACO* (*MdACO1–4*) genes have been reported^8,9^ and these genes appear to be operating similarly in the two systems for ethylene production. *MdACS1* is considered a system 2 gene; and its expression is highly correlated with the ethylene production burst in ripening apples. There are two alleles for the *MdACS1* gene, *MdACS1-1* and *MdACS1–2*, and the former is often associated with high ethylene production while the latter with lower ethylene production during fruit ripening.^10–14^ This observation has led to a marker-assisted selection strategy emphasizing on selection for allelotype (see Discussion for usage of term ‘allelotype’) *MdACS1–2/2* for long-shelf life apples.^15^ Indeed, some evidence suggests that modern apple-breeding practice has unintentionally favored selection for the *MdACS1–2* allele in commercial apple cultivars,^16^ presumably for fruit of low ethylene and long-shelf life.

However, early-ripening cultivars showed faster fruit softening, regardless of their *MdACS1* allelotypes.^10^ This is consistent with the observation that the polygalacturonase gene (*MdPG1*) involved in softening of fruit flesh is expressed irregularly among apple cultivars of identical *MdACS1* allelotypes.^12^ Therefore, there are other factors also affecting fruit shelf life in addition to *MdACS1*. Interestingly, findings in a recent report have suggested that allele variations of another *ACS* gene (U73816),^17^ designated *MdACS3a* (AB243060), are an essential factor regulating apple fruit ripening and shelf life.^18^ There are two natural mutant alleles of the wild-type allele *MdACS3a*: One is the functional null allele *MdACS3a-G289V*, arising from a point mutation that leads to an amino-acid substitution from G_289_ to V_289_ at an active region for the MdACS3A enzyme activity, resulting in a functionally inactive enzyme. In melon, a similar point mutation in a conserved active region of an *ACS* gene led to andromonoecy, a common sexual system in angiosperms characterized by carrying both male and bisexual flowers.^19^ This is an excellent example demonstrating that point mutations in conserved active regions of an ACS enzyme could confer a major phenotypic variation in plants. The other, a transcriptionally null allele *Mdacs3a*, is characterized by non-detectable mRNA. Moreover, combinations of *Mdacs3a* and *MdACS3a-G289V* alleles, regardless of whether they are homozygous or heterozygous, are highly associated with lower ethylene production and long-shelf life. In the six apple varieties/selections of the two null alleles studied, all showed low ethylene production and long-shelf life, irrespective to their *MdACS1* allelotypes and early, mid or late physiological maturation dates.^18^ Furthermore, the expression of *MdACS3a* is fruit tissue specific and detectable only during the transition from system 1 to 2 ethylene biosynthesis.^8,9,18^ These observations suggest that *MdACS3a* acts as a main regulator for the transition, and is thereby crucial in regulating the fruit-ripening process.^18^

In a more recent report, however, the allelotypes of *MdACS3a* were demonstrated to affect the ripening initiation of late-maturing cultivars only, but not the early- or mid-maturing cultivars.^20^ To better assess the roles of *MdACS1* and *MdACS3a*, two approaches were taken in this study. The first approach was to estimate the allelotypic effect of the two genes by evaluating fruit ethylene production levels and softening rates in 97 diverse *Malus* accessions and 34 progeny from 2 controlled crosses. The second approach was to examine how variations in their allelotypic effect were associated with the frequency changes of the *MdACS1* and *MdACS3a* alleles in *M. domestica* and *M. hybrid* as compared with those in *M. sieversii*, the major progenitor species of domestic apples, in 952 *Malus* accessions covering 53 *Malus* species. Allelotyping (see Discussion for usage of term ‘allelotyping’) of *MdACS1* and *MdACS3a* was conducted using an existing marker for *MdACS1* and two CAPS (cleaved amplified polymorphic sequence) markers specifically developed here to detect alleles *ACS3a-G289V* and *Mdacs3a*.

## Materials and methods

### Plant materials

Two sets of *Malus* accessions were used in this study, which have been planted and maintained in the *Malus* germplasm repository of the US Department of Agriculture (USDA) in Geneva, New York. The first set included a total of 952 accessions, covering 53 *Malus* species (Supplementary Table S1). Among them, *Malus domestica* of 508 accessions, *M. hybrid* (the breeding selections derived from crosses between *M. domestica* and other *Malus* species) of 146 and *M. sieversii* (the major progenitor species of *M. domestica*) of 78 were most commonly represented (Supplementary Table S1). The second set comprised 34 half-sib progeny selected from 2 interspecific crosses GMAL4592 (‘Royal Gala’×PI613978) and GMAL4593 (‘Royal Gala’×PI613981). ‘Royal Gala’, a widely grown apple cultivar (*M. domestica*), has an allelotype *MdACS1–2/2* and *MdACS3a/MdACS3a-G289V* for genes *MdACS1* and *MdACS3a*, respectively. PI613978 and PI613981 are among the elite selections of *M. sieversii* collected from Kazakhstan,^21^ and they have the same allelotypes for the two ACS genes, that is, *MdACS1-1/1* and *MdACS3a/MdACS3a-G289V*. Population GMAL4592 was used in one of our previous studies.^22^ Both GMAL4592 and GMAL4593 were planted on their own seedling roots in 2004.

### Measurements of fruit ethylene production and firmness

Fruit ethylene production and flesh firmness were measured for 97 of 952 *Malus* accessions in the first set and the 34 half-sib progeny in the second set as described previously.^23^ Briefly, for each accession, at least 25 fruits were harvested at a target maturity level as determined by the starch index of 4–6 according to the Cornell Starch Chart.^24^ The 25 fruits were evenly divided into 5 groups and were stored for 0, 5, 10, 15 and 20 days at room temperature (20–25 °C), respectively. Each fruit was weighed then enclosed in a gas-tight container (1.2 l) and kept for 1 h at room temperature. One milliliter of gas was sampled from the headspace in the container using a BD syringe (No. 309602, BD, Franklin Lakes, NJ, USA). The gas sample’s ethylene concentration was measured with a gas chromatograph HP 5890 series II (Hewlett-Packard, Palo Alto, CA, USA) equipped with a flame ionization detector. Before the gas samples were assayed, the gas chromatograph was calibrated with standard ethylene gas (NO. 34489, Restek, Bellefonte, PA, USA) at a series of concentrations—0.01, 0.1, 0.5, 1, 5, 10 and 100 p.p.m.—to obtain the linear relation between ethylene peak area and concentration. The fruit ethylene production was calculated with the following formula:

$$E=[C_{2}H_{4}]\times \left(V_{1}-V_{2}\right)/W/T$$

Where *E* stands for fruit ethylene production rate in nanoliter per gram of fresh weight per hour (nL g^−1^ h^−1^), [C_2_H_4_] for ethylene concentration in p.p.m., *V*_1_ for the volume of container in mL, *V*_2_ for the volume of fruit in mL equivalent to fresh weight (*W*) in grams and *T* stands for the time in hours kept in the container.

Fruit flesh firmness was measured using a penetrometer (Fruit Tester, Wagner FTK100, Greenwich, CT, USA) with a probe of 11 mm in diameter. The probe tip was pressed vertically into the fruit pulp (after skin-disc removal) to a depth of 10 mm. For larger fruits, four skin discs were removed from opposite sides of each fruit along the equator, and for smaller fruits, three skin discs were removed at roughly equal distance. The firmness readings were expressed in kg cm^−2^, and firmness loss was measured by the percentage (%) of firmness reduced at days 5 to 20 as compared with the firmness at day 0. After the firmness was measured, fruits were sliced in half along the equator, dipped into a iodine-potassium iodide (I_2_-KI) solution, and then allowed the staining reaction for >1 min before reading Cornell Starch Index.^24^

### Allelotyping of *MdACS1* and *MdACS3a*

Allelotyping of *MdACS1* was conducted with marker ACS1 using primers ACS1–5F/R (Supplementary Table S2) as reported previously.^10,15^ However, allelotyping of *MdACS3a* was accomplished with two CAPS markers developed in this study using an online tool for identifying appropriate restriction enzymes^25^ (see Results). These two markers, named CAPS_866_ and CAPS_870_, were capable of detecting the functional null allele *MdACS3a-G289V* and the transcriptional null allele *Mdacs3a*, respectively. In practice, the same primers ACS3a-289F/R (Supplementary Table S2) were used for PCR to amplify the targeted DNA fragment for both CAPS_866_ and CAPS_870_. PCRs were performed with 35 cycles of 94 °C for 30 s, 58 °C for 30 s, 72 °C for 1 min, with an initial 94 °C for 5 min and a final extension of 72 °C for 10 min. Each PCR reaction mix was set in 10 μL containing 20 ng genomic DNA, 0.2 mm each dNTP, 0.5 μm of each primer, 2.5 mm MgCl_2_, 2 μL 5× PCR Colorless GoTaq Reaction Buffer and 1 U of GoTaq DNA polymerase (Promega, Madison, WI, USA). To detect alleles *MdACS3a-G289V* and *Mdacs3a*, the PCR products were restricted with enzymes *Bst*NI and *Taq*^α^I (New England Biolabs, Ipswich, MA, USA) following the manufacturer’s instruction, respectively. The restricted PCR products were assayed by electrophoresis on 1.5% agarose gel and then stained with ethidium bromide for visualization and documentation as described previously.^22^

### Sanger DNA sequencing

The PCR products amplified by primers ACS3a-289F/R (Supplementary Table S2) were directly sequenced using a DNA Sequencer ABI3730XL (Applied Biosystems, Foster City, CA, USA) at the Cornell University Biotechnology Resource Center (Ithaca, NY, USA). The reverse PCR primer ACS3a-289R was used for DNA sequencing. DNA sequence analyses were performed using software Sequencher 5.2 (Gene Codes Corporation, Ann Arbor, MI, USA).

### Statistical analysis

Pearson’s correlation analysis and one-way analysis of variance (ANOVA) of ethylene production and fruit firmness were conducted with software JMP Pro 10.0 (SAS institute, Cary, NC, USA). Significance levels in comparison of the means were determined by *P*<0.05 (Student’s *t*-test).

## Results

### Evaluation of fruit ethylene production and softening

**Figure 1:**
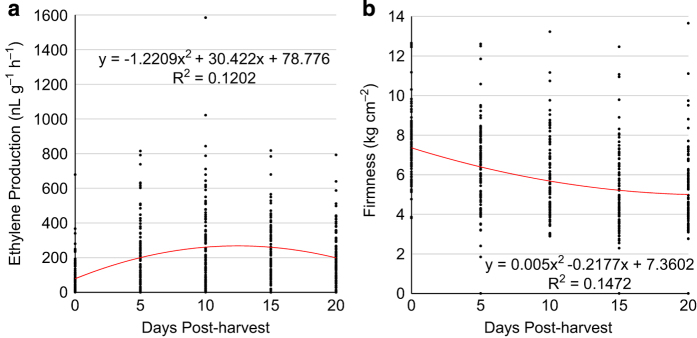
Evaluation of fruit ethylene production (**a**) and firmness (**b**) in 97 *Malus* accessions during a 20-day post-harvest period under room temperature. The trend lines (curves in red) and the associated equations and coefficient of determination (R^2^) are presented.

Fruit ethylene production and softening were evaluated in 97 of 952 *Malus* accessions (Supplementary Tables S1 and S3). Their mature date was determined by Cornell starch index, which had a mean 5.5±1.4 at harvest. The 97 accessions varied widely not only in maturity date (from 16 August to 8 November 2011; Supplementary Figure S1a) and fruit weight (25.1–303.8 g, Supplementary Figure S1b), but also in ethylene production and firmness at harvest (day 0) and during the 20-day post-harvest period (Figures 1a and b). At day 0, for example, the ethylene levels ranged from 0.7 nL g^−1^ per h of PI588844 (‘Fuji’, *M. domestica*) to 679.3 nL g^−1^ per h of PI619168 (an accession of *M. sylvestris*), and fruit firmness varied from 3.8 kg cm^−^^2^ of PI589572 (E14–32, *M. hybrid*) to 12.7 kg cm^−^^2^ of PI589478 (‘Novosibirski Sweet’, *M. domestica*). Despite being highly variable, a trend line of bivariate function could be fit for fruit ethylene production (r^2^=0.120, *P*<0.0001, Figure 1a) and fruit firmness (r^2^=0.147, *P*<0.0001, Figure 1b).

The trend line of fruit ethylene showed a peak between days 10 and 15, which was largely a reflection of the mean fruit ethylene levels 75.5±100.5, 207.3±193.9, 272.8±249.6, 247.0±170.8 and 217.3±146.5 (nL g^−1^ h^−1^) at days 0, 5, 10, 15 and 20, respectively (Figure 1a). A majority (59/97, 60.8%) of the 97 *Malus* accessions reached their peak ethylene day at day 10 (25 accessions) or day 15 (34 accessions) while 2, 16 and 20 accessions topped their ethylene production at days 0, 5 and 20 (Supplementary Figure S1c). The peak ethylene reads were spread from 1.7 nL g^−1^ per h of PI589570 (E36-7, *M. hybrid*) at day 20 to 1022.2 nL g^−1^ per h of PI633801 (*M. sieversii*) at day 10 (Supplementary Table S3).

As expected, fruit firmness showed a continuous decreasing trend during the 20-day period (Figure 1b). This was also an approximation of the mean firmness 7.4±1.7 kg cm^−^^2^, 6.5±2.1 kg cm^−^^2^, 5.8±2.0 kg cm^−^^2^, 5.3±1.99 kg cm^−^^2^ and 5.3±1.92 kg cm^−^^2^ at days 0, 5, 10, 15 and 20, respectively. In other words, the mean fruit firmness was lost by 13.6% at day 5, 22.0% at day 10, 29.2% at day 15 and 29.0% at day 20.

Fruit ethylene production and firmness loss were significantly correlated (Table 1). The strongest correlation (r=0.564, *P*=0) was observed between ethylene at day 15 and fruit firmness loss at day 10, while the weakest (r=0.214, *P*=0.035) was between ethylene at day 10 and fruit firmness loss at day 5. Peak ethylene day (day of peak ethylene production during the 20-day post-harvest storage) was most significantly correlated with ethylene at day 5 (r=−0.479, *P*=6.9E−7), and it also significantly correlated with fruit firmness loss at day 10 (r=−0.258, *P*=0.011) and day 15 (r=−0.238, *P*=0.019) (Table 1).

### Development of allelic specific markers for *MdACS3a*

**Figure 2:**
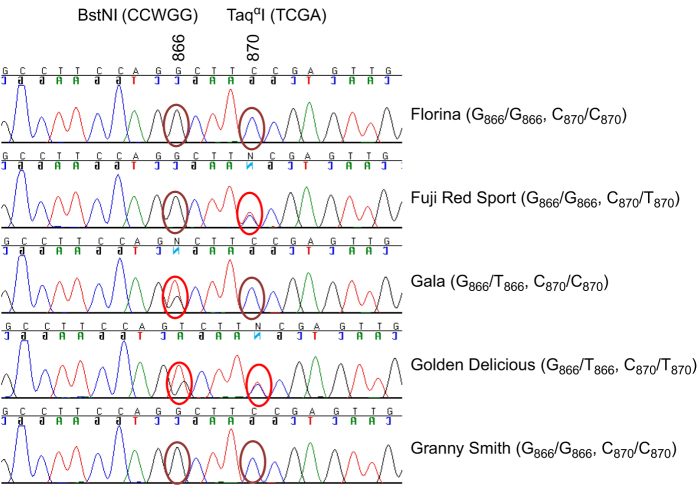
A chromatogram screenshot of the DNA sequence (partial) of *MdACS3a* encompassing SNPs G_866_/T_866_ and C_870_/T_870_ in six apple cultivars—‘Florina’, ’Fuji red sport’, ‘Gala’, ‘Golden Delicious’ and ‘Granny Smith’. The oval circles in brown and red indicate the homozygous or heterozygous status at the 866th and 870th nucleotides in the coding sequence of *MdACS3a*, respectively. The recognition sites of restriction enzymes *Bst*NI and *Taq*^α^I are provided to show that the mutation from G_866_ to T_866_ abolishes the restriction site of *Bst*NI while the mutation from C_870_ to T_870_ gives rise to a restriction site for *Taq*^α^I. The right panel shows allelotypes of *MdACS3a* as represented by the SNP alleles, where G_866_ stands for allele *MdACS3a* (wild type), T_866_ for *MdACS3a-G289V* (functional null allele), C_870_ also for allele *MdACS3a* and T_870_ for *Mdacs3a* (transcriptional null allele).

**Figure 3:**
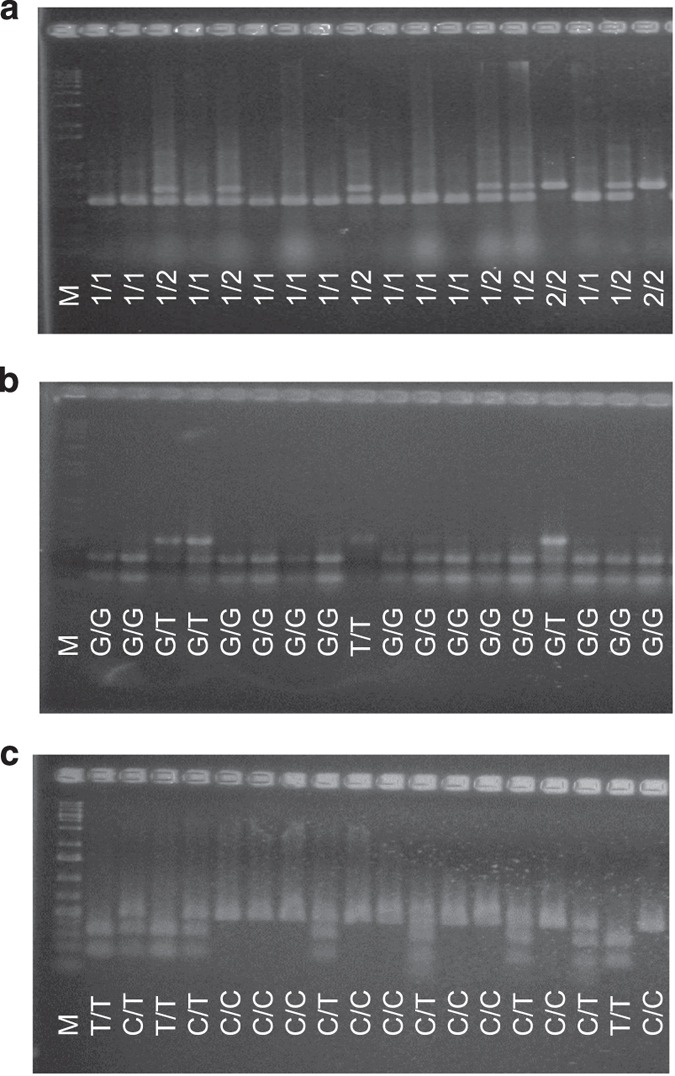
Agarose gel analyses of markers ACS1 (**a**), CAPS_866_ (**b**) and CAPS_870_ (**c**). For marker ACS1, the PCR products amplified by primers ACS1–5F/R were directly analyzed. Allelotypes *MdACS1-1/1*, *MdACS1–2/2* and *MdACS1-1/2* are denoted with ‘1/1’, ‘2/2’ and ‘1/2’, respectively. For marker CAPS_866_, the PCR products were first amplified by primers ACS3a-289F/R and then digested with enzyme *Bst*NI, which restricts the *MdACS3a* (G_866_) allele into the two lower bands. Allelotypes *MdACS3a*/*MdACS3a* (G_866_/G_866_), *MdACS3a*/*MdACS3a-G289V* (G_866_/T_866_) and *MdACS3a-G289V/G289V* (T_866_/T_866_) are noted with ‘G/G’, ‘G/T’ and ‘T/T’, respectively. For marker CAPS_870_, enzyme *Taq*^α^I restricts the *Mdacs3a* (T_870_) allele into the two lower bands. Allelotypes *MdACS3a*/*MdACS3a* (C_870_/C_870_), *MdACS3a*/*mdacs3a* (C_870_/T_870_) and *mdacs3a/mdacs3a* (T_870_/T_870_) are noted with ‘C/C’, ‘C/T’ and ‘T/T’, respectively.

The null allele *MdACS3a-G289V* is caused by a mutation from G_866_ to T_866_ at the 866th base in the coding sequence of *MdACS3a*.^18^ Based on the web-based tool for single nucleotide polymorphism (SNP) analysis,^25^ the mutation abolishes the recognition site CC_866_WGG of restriction enzyme *Bst*NI (Figure 2). To develop a CAPS marker, two primers (ACS3a-289F/R, Supplementary Table S2) were designed to amplify a DNA fragment (480 bp) covering the SNP (G_866_/T_866)_ specifically from *MdACS3a* although the three *MdACS3* member genes *MdACS3a* (AB243060), *MdACS3b* (AB243061) and *MdACS3c* (AB243062) are of high identity in their DNA sequences.^18^ The specificity of the primer pair to *MdACS3a* was confirmed by sequencing of the PCR products from 92 of the 97 *Malus* accessions (Figure 2, Supplementary Table S3). Digestion of the PCR products with *Bst*NI yielded restriction bands as expected (Figure 3b), indicating the successful development of a CAPS marker detecting SNP G_866_/T_866_, designated CAPS_866_. Therefore, allele *CAPS*_*866*_*G* represents the wild-type allele *MdACS3a* while *CAPS*_*866*_*T* stands for the functional null allele *MdACS3a-G289V*.

Development of a marker detecting the transcriptional null allele *Mdacs3a* was initially thought to be challenging as the null allele was reported not to show sequence variations from the wild-type allele.^18^ However, sequencing analysis of the PCR products amplified by primers ACS3a-289F/R in the 92 accessions (Supplementary Table S3) not only identified the expected SNP G_866_/T_866_, but also a new SNP C_870_/T_870_ (Figure 2). Importantly, this new SNP can discriminate the two alleles of *MdACS3a* in ‘Fuji’ (Figure 2), which was known of allelotype *MdACS3a*/*Mdacs3a*.^18^ Evidence from this and other studies (see Discussion) indicated that base T_870_ was associated with the *Mdacs3a* allele. Using a similar approach, another CAPS marker, named CAPS_870_, was developed to detect SNP C_870_/T_870_ using restriction enzyme *Taq*^α^I along with the same primers ACS3a-289F/R (Figure 3c). Therefore, allele *CAPS*_*870*_*C* corresponds to the wild-type allele *MdACS3a* while *CAPS*_*870*_*T* corresponds to the transcriptional null allele *Mdacs3a*.

### Effect of the allelotypes of *MdACS1* and *MdACS3a* on ethylene production and firmness loss

To evaluate the effect of the allelotypes of *MdACS1* and *MdACS3a*, the 97 *Malus* accessions were assayed with markers ACS1, CAPS_866_ and CAPS_870_ that can detect different alleles of *MdACS1* and *MdACS3a* (Figures 3a–c). As a result, marker ACS1 identified 53, 36 and 8 accessions of allelotypes of *MdACS1-1*/*MdACS1-1* (*MdACS1-1/1*), *MdACS1-1*/*MdACS1–2* (*MdACS1-1*/*2*) and *MdACS1–2*/*MdACS1–2* (*MdACS1–2/2*), respectively (Supplementary Table S3). Similarly, marker CAPS_866_ detected 75 accessions of allelotype *CAPS*_*866*_*G/CAPS*_*866*_*G* (*CAPS*_*866*_*G/G*), 18 of *CAPS*_*866*_*G/CAPS*_*866*_*T* (*CAPS*_*866*_*G/T*) and 4 of *CAPS*_*866*_*T/CAPS*_*866*_*T* (*CAPS*_*866*_*T/T*); and marker CAPS_870_ uncovered 47 accessions of allelotype *CAPS*_*870*_*C/CAPS*_*870*_*C* (*CAPS*_*870*_*C/C*), 40 of *CAPS*_*870*_*C/CAPS*_*870*_*T* (*CAPS*_*870*_*C/T*) and 10 of *CAPS*_*870*_*C/CAPS*_*870*_*T* (*CAPS*_*870*_*T/T*) (Supplementary Table S3).

**Figure 4:**
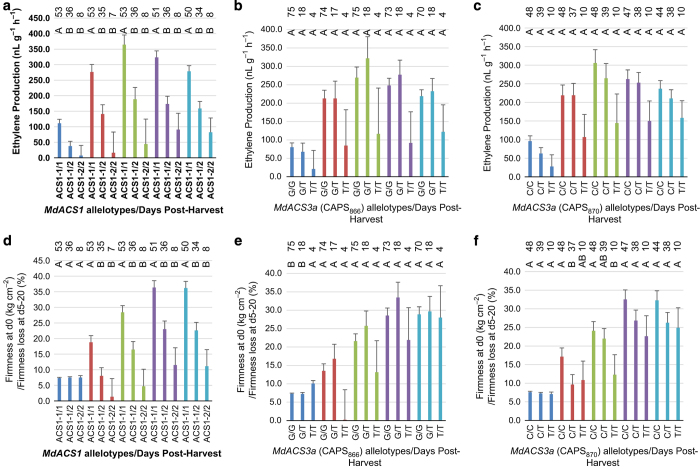
Comparison of the means of fruit ethylene production and firmness or firmness loss among allelotypes of *MdACS1* as defined by marker ACS1 (**a** and **d**), and among those of *MdACS3a* as defined by markers CAPS_866_ (**b** and **e**) and CAPS_870_ (**c** and **f**). The allelotypes are annotated similarly as those in the legend of Figure 3. Colors of column in blue, orange, green, purple and turquoise represent days 0, 5, 10, 15 and 20, respectively. The statistical tests were conducted independently within each of the five storage time points (days 0–20). Significance levels are indicated with letters (shown above the columns in the chart), where different letters indicate *P*<0.05. The numbers of accessions observed (*n*) for each allelotype are presented accordingly (shown above the letters for significance). Error bars indicate s.e.

A series of one-way ANOVA of the fruit ethylene production and fruit firmness loss over the 20-day period within each of the three allelotype groups (Figure 4) indicated that the most differences were observed among the *MdACS1* allelotypes. Allelotype *MdACS1-1/1* showed significantly higher ethylene production (days 0–20) and firmness loss (days 5–20) than *MdACS1-1/2* and *MdACS1–2/2* allelotypes, but *MdACS1-1/2* and *MdACS1–2/2* did not differ in terms of ethylene production or firmness retention (Figures 4a and d). In contrast, there were no difference among the CAPS_866_ allelotypes in fruit ethylene production and firmness loss (Figures 4b and e). Among the CAPS_870_ allelotypes, significant difference was not detected for ethylene production, but there were differences in fruit firmness loss between allelotypes *CAPS*_*870*_*C/C* and *CAPS*_*870*_*C/T* at day 5 and between *CAPS*_*870*_*C/C* and *CAPS*_*870*_*T/T* at day 10 (Figures 4c and f). This indicated that such differences in fruit firmness loss at day 5 and 10 in the CAPS_870_ allelotypes might be caused by other factors rather than their ethylene production levels.

**Figure 5:**
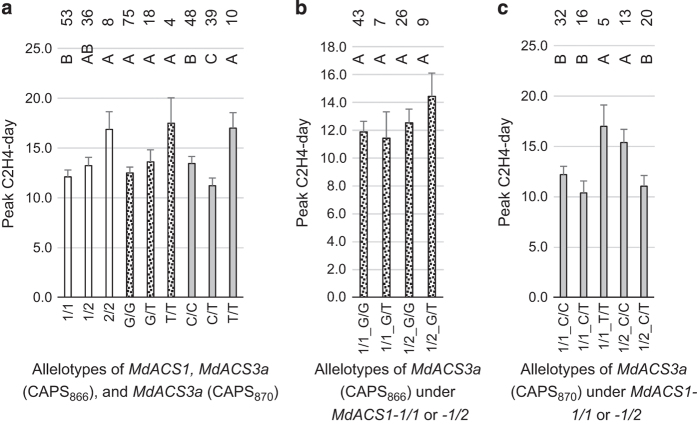
Comparison of the means of peak ethylene day among the allelotypes of *MdACS1* as defined by marker ACS1 (open column) and those of *MdACS3a* as defined by markers CAPS_866_ (dot-filled column) and CAPS_870_ (filled column) (**a**), and among the allelotypes of *MdACS3a* defined by markers CAPS_866_ (**b**) and CAPS_870_ (**c**) under the same background of *MdACS1-1/1* or *MdaCS1-1/2*. The allelotypes, significance levels and observed numbers are represented similarly as those in Figures 3 and 4.

To seek such factors, peak ethylene day, which measures ethylene peak timing, was examined (Figure 5) as this trait was negatively correlated with fruit firmness loss at day 10 (r=−0.258, *P*=0.011) although the correlation was insignificant at day 5 (r=−0.112, *P*=0.275) (Table 1). Encouragingly, the three CAPS_870_ allelotypes showed significant difference from each other, with *CAPS*_*870*_*C/T* having peaked the earliest, *CAPS*_*870*_*C/C* intermediate and *CAPS*_*870*_*T/T* the latest (Figure 5a). These data appeared to suggest that the earlier peak ethylene day of *CAPS*_*870*_*C/C* might have contributed to its greater fruit firmness loss of *CAPS*_*870*_*C/C* as compared with that of *CAPS*_*870*_*T/T* at day 10 (Figure 4f). However, the lowest fruit firmness loss of *CAPS*_*870*_*C/T* at day 5 remained to be explained. Peak ethylene day was also analyzed in the other two groups of allelotypes. In the allelotypes of *MdACS1*, *MdACS1-1*/*1* had an earlier peak ethylene than *MdACS1–2*/*2*, but showed no difference from *MdACS1-1*/*2* (Figure 5a). In the three allelotypes of CAPS_866_, no significant difference was observed (Figure 5a).

**Figure 6:**
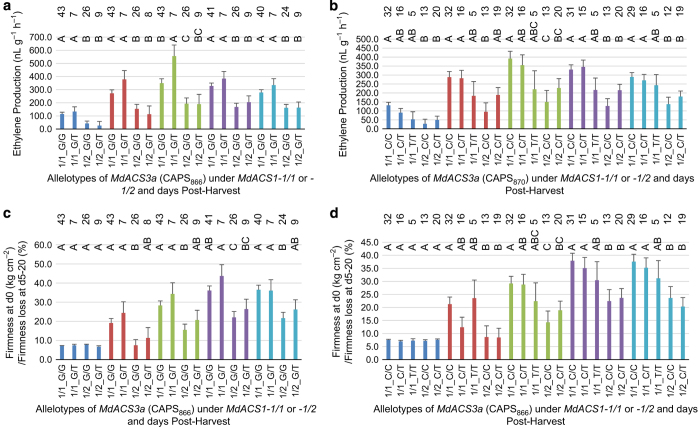
Comparison of the means of ethylene production and fruit firmness or firmness loss among the allelotypes of *MdACS3a* as defined by markers CAPS_866_ (**a** and **c**) and CAPS_870_ (**b** and **d**) under the same background of *MdACS1-1/1* or *MdaCS1-1/2*. The allelotypes, column colors, statistical tests, significance levels and observed numbers are represented similarly as those in Figures 3 and 4.

It was clear that the effect of *MdACS1* on ethylene production and fruit firmness loss was much stronger than that of *MdACS3a* (Figure 4). To see if the random presence of the *MdACS1* alleles might have obscured the detection of the effect of *MdACS3a* allelotypes (Figures 4b, c, e and f), another series of ANOVA was conducted for the *MdACS3a* allelotypes of five or more accessions (Figure 6) under the same background of *MdACS1* allelotypes *MdACS1-1*/*1* and *MdACS1-1*/*2*, which occurred in 53 and 36 of the 97 accessions (Supplementary Table S3), respectively. The third allelotype *MdACS1–2*/*2* was not included in the analysis (Figure 6) due to limited number of 8 accessions.

For CAPS_866_, the ANOVA analyses were conducted for two allelotypes *CAPS*_*866*_*G/G* and *CAPS*_*866*_*G/T* under *MdACS1-1*/*1*, as well as under *MdACS1-1*/*2* (Figures 5b and 6a,c). This allowed us to identify that allelotype *CAPS*_*866*_*G/T* produced significantly higher levels of ethylene than *CAPS*_*866*_*G/G* at day 10 under *MdACS1-1*/*1* (Figure 6a). For CAPS_870_, three allelotypes *CAPS*_*870*_*C/C*, *CAPS*_*870*_*C/T* and *CAPS*_*870*_*T/T* under *MdACS1-1*/*1* and two allelotypes *CAPS*_*870*_*C/C* and *CAPS*_*870*_*C/T* under *MdACS1-1*/*2* were analyzed (Figures 5c and 6b,d). The results showed that allelotype *CAPS*_*870*_*T/T* had significant later peak ethylene day than *CAPS*_*870*_*C/C* and *CAPS*_*870*_*C/T* under *MdACS1-1*/*1,* and *CAPS*_*870*_*C/C* had significant later peak ethylene than *CAPS*_*870*_*C/T* under *MdACS1-1*/2 (Figure 5c). There were no significant differences detected between the other allelotypes of CAPS_866_ and CAPS_870_ at a given time point (Figures 5b and 6a–d). These observations suggested that the direct effect of *MdACS3a* on ethylene production and firmness loss was limited, but its effect on peak ethylene day was clearly detectable through allele *Mdacs3a* (*CAPS*_*870*_*T/T*).

The analyses also provided information regarding the effect of *MdACS1* under the same background of CAPS_866_ (Figures 5b and 6a,c) or CAPS_870_ (Figures 5c and 6b,d) allelotypes. As expected, allelotype *MdACS1-1*/*1* had higher ethylene production (Figures 6a and c) and more firmness loss (Figures 6b and d) than *MdACS1–2/2*, but had similar peak ethylene day as *MdACS1-1*/*2* (Figures 5b and c) except under the *CAPS*_*870*_*C/C* background (Figure 5c). These results suggested that the effect of *MdACS1* on peak ethylene day was insignificant under the same background of *MdACS3a*, which was in disagreement with the observation that the effect of *MdACS1* on peak ethylene day was significant when the background of *MdACS3a* was not considered (Figure 5a).

Since the *MdACS3a* allelotype *CAPS*_*866*_*T/T* (*MdACS3a-G289V/G289V*) was present only in 4 of 97 accessions, the 2 controlled crosses GMAL4592 and GMAL4593 segregating for *CAPS*_*866*_*T/T* under the same background of *MdACS1-1*/*2* were used for better analysis. In total, 17 progeny of allelotype *CAPS*_*866*_*G/G* (*MdACS3a/MdACS3a*) and another 17 of *CAPS*_*866*_*T/T* were similarly evaluated for ethylene production and fruit firmness loss. ANOVA analysis indicated that there were no significant differences between the two allelotypes *CAPS*_*866*_*G/G* and *CAPS*_*866*_*T/T* in ethylene production and fruit firmness loss, nor in peak ethylene day from day 0 to day 20 (Supplementary Figures 2a–c), suggesting that no effect of allelotype *CAPS*_*866*_*T/T* (*MdACS3a-G289V/G289V*) was detectable in this study.

### Allelotyping of *MdACS1* and *MdACS3a* in a large set of *Malus* accessions

**Figure 7:**
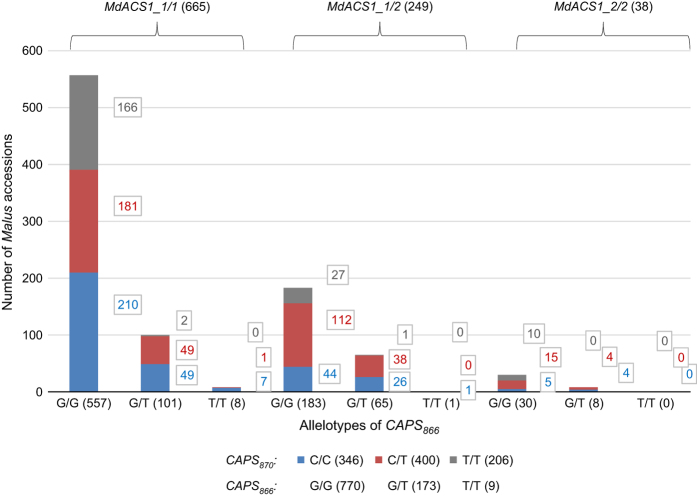
Allelotyping of *MdACS1* and *MdACS3a* using markers ACS1, CAPS_866_ and CAPS_870_ in 952 *Malus* accessions. The numbers in parentheses stand for the total or subtotal number of *Malus* accessions in an allelotype proximately annotated. The allelotypes are represented similarly as those in Figure 3.

**Figure 8:**
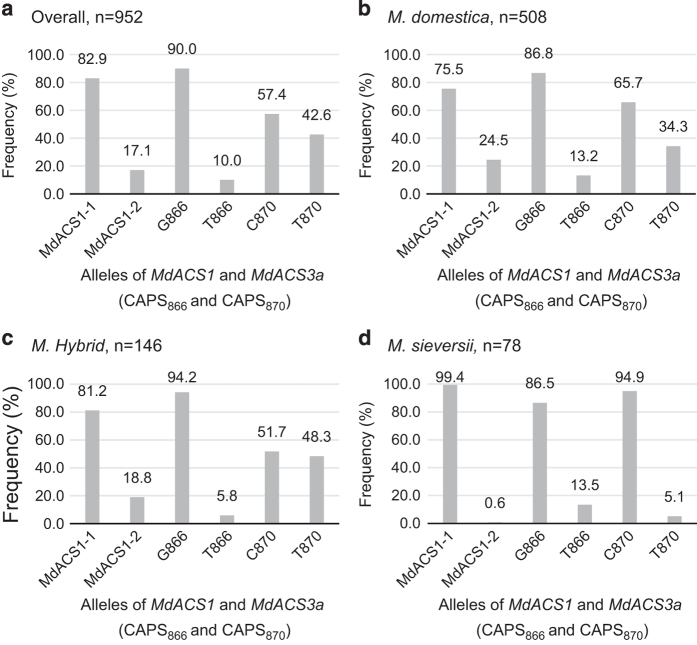
Frequency of the *MdACS1* and *MdACS3a* alleles as defined by markers ACS1, CAPS_866_ and CAPS_870_ in all the 952 *Malus* accessions (**a**), *M. domestica* (**b**), *M. hybrid* (**c**) and *M. sieversii* (**d**).

Additional 855 *Malus* accessions were surveyed with markers ACS1, CAPS_866_ and CAPS_870_, leading to a total of 952 *Malus* accessions allelotyped (Figure 7, Supplementary Table S1). The data showed that the three allelotypes *MdACS1-1/1*, *MdACS1-1/2* and *MdACS1–2/2* were of 665, 249 and 38 accessions, the allelotypes *CAPS*_*866*_*G/G*, *CAPS*_*866*_*G/T* and *CAPS*_*866*_*T/T* were of 770, 173 and 9 accessions, and the allelotypes *CAPS*_*870*_*C/C*, *CAPS*_*870*_*C/T* and *CAPS*_*870*_*T/T* were of 346, 400 and 206 accessions, respectively. Estimating the allele frequency in the 952 accessions revealed alleles *MdACS1-1* and *MdACS1–2* of 82.9% and 17.1%, *CAPS*_*866*_*G* and *CAPS*_*866*_*T* of 90.0% and 10.0%, and *CAPS*_*870*_*C* and *CAPS*_*870*_*T* of 57.4% and 42.6%, respectively (Figure 8a).

To investigate whether and how human selection might have favored or repressed these alleles, their frequency in the most represented species *M. domestica* (508 accessions), *M. hybrid* (146) and *M. sieversii* (78), which collectively accounted for 76.9% of the 952 accessions (Supplementary Table S1), were independently estimated (Figures 8b–d). In comparison with *M. sieversii, M. domestica* and *M. hybrid* showed the largest allele frequency increases for alleles *MdACS1–2* (from 0.6% to 18.8–24.5%) and *CAPS*_*870*_*T* (from 5.1% to 34.3–48.3%), or decreases for allele *MdACS1-1* (from 99.4% to 81.2–75.5%) and *CAPS*_*870*_*C* (from 94.9% to 65.7–51.7%), but minimal changes for the *CAPS*_*866*_*G* (from 86.5% to 86.8–94.2%) and *CAPS*_*866*_*T* (from 13.5% to 13.2–5.8%) alleles (Figures 8b–d). These results suggested that apple-breeding practice may have selected for alleles *MdACS1–2* and *CAPS*_*870*_*T* (*Mdacs3a*), against alleles *MdACS1-1* and *CAPS*_*870*_*C*, and is neutral for alleles *CAPS*_*866*_*G* and *CAPS*_*866*_*T* (*MdACS3a-G289V*). Such human selection for alleles *MdACS1–2* and *Mdacs3a* supported their observed significant effect on reduced or delayed ethylene production. Meanwhile, the minimal changes in the frequency of allele *MdACS3a-G289V* reinforced the unfound effect of this allele on ethylene.

## Discussion

### The effect of *MdACS1* and *MdACS3a* and beneficial alleles

The allelic effect of *MdACS1* on fruit ethylene production and softening was significant and detectable at nearly all time points tested during the 20-day post-harvest period in the 97 *Malus* accessions. This was consistent with the critical role of *MdACS1* reported in many other studies.^10–16,26–29^ Since the allele frequency of *MdACS1–2* was 24.5% in *M. domestica*, 18.8% in *M. hybrid* and only 0.6% in *M. sieversii* (Figure 8), which is the major progenitor species of domestic apples, artificial selection has clearly favored *MdACS1–2* over *MdACS1-1*. In fact, such allele preference of *MdACS1–2* over *MdACS1-1* was even reported within *M. domestica* when the frequencies of the two alleles in apple cultivars were plotted against their time of introduction.^16^ These observations are in accordance with the finding that allele *MdACS1–2* is a beneficial allele associated with low ethylene and slow softening (Figures 4 and 6).

*MdACS3a* was regarded a main regulator for ethylene production transition from system 1 to 2.^18^ The gene was also similarly shown to be an accelerator^30^ or an inducer^31^ of apple fruit ripening based on its gene expression timing and patterns in apple cultivars of varying ethylene levels and softening rates. In this study, such roles of *MdACS3a* were also detected through examining the allelic effect of *Mdacs3a* (*CAPS*_*870*_*T*) on peak ethylene day, which reflects the timing of the climacteric ethylene burst. For example, under the same background of *MdACS1-1*/*1*, allelotype *Mdacs3a*/*Mdacs3a* (*CAPS*_*870*_*T/T*) showed a significant delay in peak ethylene day when compared with what was observed for allelotypes *MdACS3a/MdACS3a* (*CAPS*_*870*_*G/G*) and *MdACS3a/Mdacs3a* (*CAPS*_*870*_*G/T*) (Figure 5c). Moreover, the allele frequency of *Mdacs3a* (*CAPS*_*870*_*T*) was 34.3% in *M. domestica* and 48.3% in *M. hybrid*, a dramatic increase from the corresponding frequency of 5.1% in *M. sieversii*, indicating a strong human selection for allele *Mdacs3a*, presumably for the benefit of delayed ethylene production. Taken together, these data support the regulatory role of *MdACS3a* in ethylene production transition in apple fruit.

However, the allelic effect of *MdACS3a-G289V* on fruit ethylene production, softening and peak ethylene day was shown to be insignificant in the 97 Malus accessions, as well as in the 34 progeny from the 2 controlled crosses segregating for allelotype *MdACS3a-G289V/G289V* (*CAPS*_*866*_*T/T*) under the same background of *MdACS1* allelotype. Furthermore, the allele frequency of *MdACS3a-G289V* (*CAPS*_*866*_*T*) was 13.5% in *M. sieversii*, 13.2% in *M. domestica* and 5.8% in *M. hybrid*, providing no evidence that *MdACS3a-G289V* (*CAPS*_*866*_*T*) has been enriched in response to selection. These results were surprising as *MdACS3a-G289V* was shown to be a functional null allele of *MdACS3a*.^18^ In a previous study, the two null alleles *MdACS3a-G289V* (*CAPS*_*866*_*T*) and *Mdacs3a* (*CAPS*_*870*_*T*) were concluded to affect the ripening initiation only in late-season apple cultivars, but not in early- or mid-season ones.^20^ Such discrepancy in different studies regarding the roles of the two null alleles of *MdACS3a*, particularly *MdACS3a-G289V*, calls for further investigations into the role of *MdACS3a-G289V*. Nevertheless, alleles *MdACS1–2* and *Mdacs3a* (*CAPS*_*870*_*T*) are clearly demonstrated to be beneficial for breeding apples of low or delayed ethylene profiles in this study, a first effort that simultaneously assessed the roles of *MdACS1* and *MdACS3a* in fruit ethylene production and softening in highly diverse *Malus* materials.

### Markers ACS1, CAPS_866_ and CAPS_870_

The assessment of the roles of *MdACS1* and *MdACS3a* in apple fruit ethylene production and softening largely relied on the previously developed marker ACS1^10,11^ and the two markers CAPS_866_ and CAPS_870_ developed in this study. Since CAPS_866_ directly detects the mutation SNP G_866_/T_866_, CAPS_866_ is an unequivocal marker for identifying the functionally null allele *MdACS3a-G289V*.^18^ Marker CAPS_870_ detects SNP C_870_/T_870_ that does not correspond to a change in the encoding amino acid, that is, CAPS_870_ detects a silent mutation in *MdACS3a*. Regardless of the nature of SNP C_870_/T_870_, T_870_ is a genetic signature for allele *Mdacs3a* as the mutation was identified in ‘Fuji’, the very source from which the transcriptional null allele *Mdacs3a* was originally defined.^18^ Based on the genomic DNA sequences from ‘Fuji’, alleles *MdACS3a* (JF833309) and *Mdacs3a* (JF833309) differ by 14 nucleotides, and of these, only 4 were within the coding sequence.^20^ Sequencing of the 92 *Malus* accessions in this study indicated that SNP C_870_/T_870_ is authentic and varying only between 2 nucleotides C_870_ and T_870_ (Figure 2, Supplementary Table S3). These data strongly support that CAPS_870_ is a reliable marker for detecting allele *Mdacs3a*. Since both CAPS_866_ and CAPS_870_ detect the characterized SNPs in the coding sequence of *MdACS3a* and can be simply performed by electrophoresis on agarose gels, the two markers are readily applicable for marker-assisted selection in apple breeding.

Since SNP C_870_/T_870_ is located only four bases downstream of SNP G_866_/T_866_, markers CAPS_866_ and CAPS_870_ were once considered to be used as a single marker in this study. However, such usage would lead to an ambiguous scenario for allelotype G_866_T_866_/C_870_T_870_ as it could be formed by a combination either between gametes G_866_T_870_ and T_866_C_870_ or between gametes G_866_C_870_ and T_866_T_870_. To avoid such possible uncertainty, the two markers were used independently.

Previously, an SSR marker targeting at the promoter region of *MdACS3a* was developed and used to allelotype *MdACS3a* in 103 apple varieties.^20^ It was shown that three alleles (331, 353, and 359 bp) of the SSR marker corresponded to the wild-type allele *MdACS3a* (that is, *MdACS3a-1* in ref. 20), two alleles (333 and 335 bp) to *Mdacs3a* (that is, *MdACS3a-*2) and one allele (361 bp) to *MdACS3a-G289V* (that is, *MdACS3a-1V*). This makes the corresponding relationship between the SSR marker alleles and the *MdACS3a* alleles somewhat indirect and inconvenient. Since the size of the SSR marker alleles frequently differ by 2 bp, an automatic DNA sequencer-based detection system is necessary, thereby requiring more sophisticated handling and analysis, compared with the agarose gel-based markers CAPS_866_ and CAPS_870_. However, identical allelotypes were observed for all 19 apple cultivars used by co-insistence in both studies (Supplementary Table S4), suggesting that the SSR marker and the 2 CAPS markers are useful for allelotyping of *MdACS3a*. As expected, identical allelotypes for *MdACS1* were also obtained for the 19 common apple cultivars between these 2 studies (Supplementary Table S4).

It should be mentioned that two degenerated CAPS (dCAPS) markers were developed to confirm alleles *Mdacs3a* and *MdACS3a-G289V* in cDNA, but the two dCAPS markers were not used for allelotyping the *MdACS3a* alleles.^20^ Therefore, the applicability of the dCAPS markers is unknown in diverse apples.

### Utility of the data

Of the 952 *Malus* accessions, 97 were evaluated for their fruit ethylene production and softening at 5 time points over a 20-day post-harvest period (Supplementary Table S3). Although most accessions seemed to have predictable ethylene-regulated post-harvest behaviors, ‘Virginia Gold’ (PI588778, *M. domestica*) was unusual as it had minimal firmness loss (comparable to ‘Fuji’) during the 20-day storage while producing high levels of ethylene (comparable to ‘Golden Delicious’). This suggested that the slow softening (long-shelf life) character of ‘Virginia Gold’ is likely less dependent on ethylene production. More importantly, 'Virginia Gold' has also been shown with an excellent storability.^32^ To understand the lack of ethylene-related softening in ‘Virginia Gold’, several preliminary experiments have been initiated by the authors. In melon, it was reported that flesh softening involved both ethylene-dependent and -independent components.^33^ In tomato, the ethylene-independent aspects of fruit ripening were evidenced to be regulated by the FRUITFULL homologs.^34^ It is possible that investigating fruit softening independent of or less dependent on ethylene production would lead to new knowledge for better understanding of the apple fruit-ripening process, promising an interesting research area in apple post-harvest biology.

In addition, the data set of allelotypes for genes *MdACS1* and *MdACS3a* generated in the 952 *Malus* accessions would be useful for other future studies involving *MdACS1* and *MdACS3a*, which are the only 2 apple ACS genes known to be expressed specifically in fruit and associated with apple fruit ethylene production and firmness.^8,9,13^ The data set, together with three markers ACS1, CAPS_866_ and CAPS_870_, would be also useful for planning new crosses for developing improved apples with low ethylene and reduced loss of firmness.

### Usage of terms allelotype and allelotyping

Term allelotype is defined as ‘the frequency of alleles in a breeding population.’ according to 'A Dictionary of Genetics'.^35^ In this study, allelotype is referred to the allele composition at a specific gene locus, that is, *MdACS1* or *MdACS3a*, in individual accessions, highly similar to term ‘genotype’ for a given DNA marker. Such usage of allelotype represents a drift from or an expansion for the original definition of allelotype defined in the dictionary. However, the usage offers convenience for describing allele composition at a specific gene locus. Indeed, such usage has been adapted already in literature.^14,18,20^

The definition for term allelotyping in *'*'Encyclopedia of Genetics, Genomics, Proteomics, and Informatics'^36^ reads ‘Allelotyping is the determination of the spectrum and frequency of allelic variations in a population.’ The usage of allelotyping in this study is largely covered by the definition, but an extension to include activities for determining allelotype (allele composition at a specific gene locus) is also practiced.

### Conclusions

A substantial effort to simultaneously assess the roles of *MdACS1* and *MdACS3a* in fruit ethylene production and softening in diverse *Malus* materials is presented in this study. The most relevant findings include: (1) *MdACS1* had much greater direct influence on fruit ethylene production and softening than *MdACS3a*. (2) Allele *MdACS1–2* was associated with low ethylene and slow softening while *MdACS1-1* with high ethylene and rapid softening. (3) Under the same background of *MdACS1* allelotypes, the transcriptional null allele *Mdacs3a,* rather than the functional null allele *ACS3a-G289V,* significantly delayed the time required to reach the climacteric ethylene peak. (4) Alleles *MdACS1–2* and *Mdacs3a*, but not *ACS3a-G289V*, were highly enriched in *M. domestica* and *M. hybrid* when compared with those in the *M. sieversii*. Overall, this study provides important information as to which alleles of *MdACS1* and *MdACS3a* are beneficial for low and delayed ethylene production and how these beneficial alleles can be selected for apple improvement.

## Figures and Tables

**Table 1:** Correlation coefficients between fruit ethylene production and firmness or firmness loss in 97 *Malus* accessions^a^

	*C*_*2*_*H*_*4*_*_d0*	*C*_*2*_*H*_*4_*_*d5*	*C*_*2*_*H*_*4*_*_d10*	*C*_*2*_*H*_*4_*_*d15*	*C*_*2*_*H*_*4*_*_d20*	*Firmness d0 (kg cm^−^^2^)*	*Firmness loss_d5 (%)*	*Firmness loss_d10 (%)*	*Firmness loss_d15 (%)*	*Firmness loss_d20 (%)*	*Peak C*_*2*_*H*_*4*_ *day*^b^
C_2_H_4__d0	1.000**										
C_2_H_4__d5	0.434**	1.000**									
C_2_H_4__d10	0.410**	0.695**	1.000**								
C_2_H_4__d15	0.353**	0.734**	0.839**	1.000**							
C_2_H_4__d20	0.265**	0.733**	0.695**	0.871**	1.000**						
Firmness d0 (kg cm^−^^2^)	−0.208*	−0.261**	−0.164	−0.239*	−0.220*	1.000**					
Firmness loss_d5 (%)	0.324**	0.484**	0.214*	0.334**	0.431**	−0.198	1.000**				
Firmness loss_d10 (%)	0.345**	0.538**	0.493**	0.564**	0.478**	−0.092	0.704**	1.000**			
Firmness loss_d15 (%)	0.284**	0.446**	0.421**	0.481**	0.442**	−0.104	0.699**	0.863**	1.000**		
Firmness loss_d20 (%)	0.306**	0.453**	0.388**	0.481**	0.438**	−0.041	0.596**	0.828**	0.853**	1.000**	
Peak C_2_H_4_ day^b^	−0.229*	−0.479**	−0.402**	−0.281**	−0.211*	0.219*	−0.112	−0.258*	−0.238*	−0.190	1.000**

a Fruit firmness loss was measured in a 20-day post-harvest period under room temperature.

b Peak C_2_H_4_ (ethylene) day: day of peak ethylene production during the 20-day post-harvest storage; signs '*' and '**' stand for significance levels exceeding *P*=0.05 (*r*=0.1996, *n*=97) and *P*=0.01 (*r*=0.2603, *n*=97), respectively.
