# Supplementary material for: Assessing the allelotypic effect of two aminocyclopropane carboxylic acid synthase-encoding genes MdACS1 and MdACS3a on fruit ethylene production and softening in Malus
Source: Hortic Res. 2016 May 18;3:16024–. doi: 10.1038/hortres.2016.24 (PMC4870385; doi:10.1038/hortres.2016.24)
Supplement: Supplementary Legends [file hortres201624-s1.doc]

**Supplementary information**

1. Figure S1. Distribution of fruit maturity/harvest date (**a**), fruit weight (**b**), and peak ethylene day (**c**).
2. Figure S2. Comparison of the means of ethylene production (**a**), fruit firmness (**b**) and peak ethylene day (**c**) among allelotypes of *MdACS3a*as defined by marker CAPS_866_ under the same background of *MdACS1-1/2*in the 34 progeny of crosses GMAL4592 and GMAL4593.Allelotypes *MdACS3a*/*MdACS3a* (G_866_/G_866_) and *MdACS3a-G289V/G289V* (T_866_/T_866_) are noted with ‘G/G’ and ‘T/T’, respectively. Colors of column in blue, orange, green, purple and turquoise represent d0, d5, d10, d15 and d20, respectively. Significance levels are indicated with letters, where different letters indicate p<0.05. The numbers of accessions observed (n) for each allelotype are presented accordingly. Error bars indicate standard errors.
3. Table S1. List of 952 *Malu*s accessions allelotyped with markers ACS1 and CAPS_866_ and CAPS_870_.
4. Table S2. Allele specific primers for genes *MdACS1* and *MdACS3a*
5. Table S3. Evaluation of fruit ethylene production and firmness in a subset of 97 *Malus* accessions
6. Table S4. Comparison of the *MdACS1* and *MdACS3a* allelotypes in *Malus* accessions used in both Bai et al. and this study.
