# Supplementary figures and images for: Assessing the allelotypic effect of two aminocyclopropane carboxylic acid synthase-encoding genes MdACS1 and MdACS3a on fruit ethylene production and softening in Malus
Source: Hortic Res. 2016 May 18;3:16024–. doi: 10.1038/hortres.2016.24 (PMC4870385; doi:10.1038/hortres.2016.24)

Figure S1

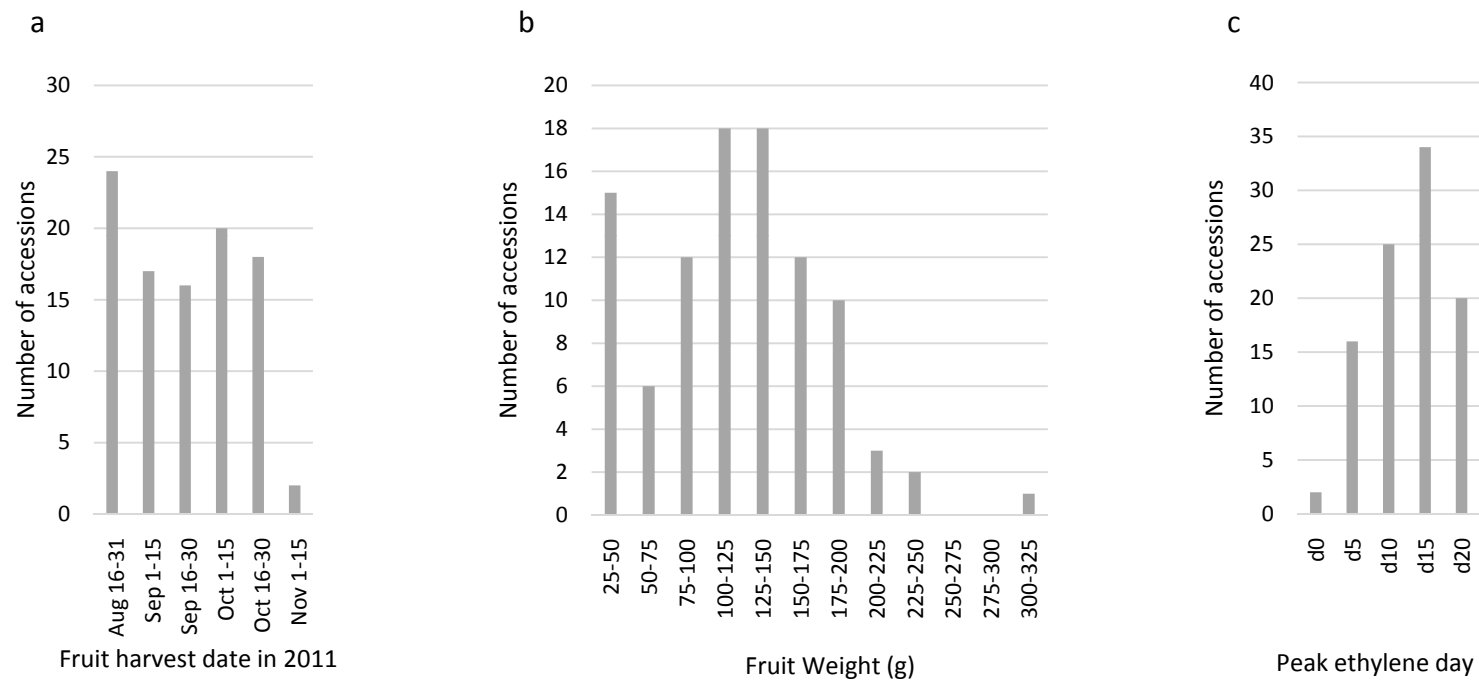

Figure S2

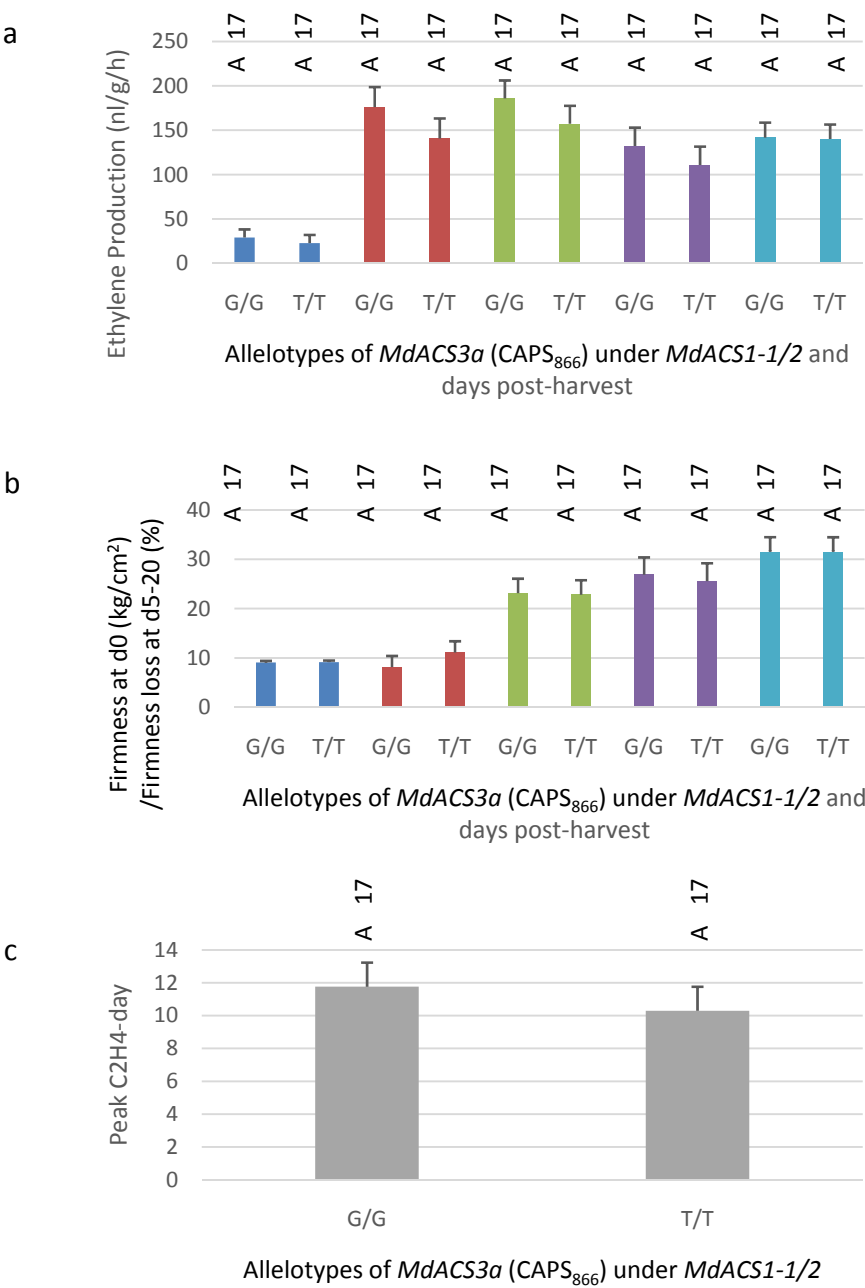

Supplement: Supplementary Figures S1–S2 [file hortres201624-s2.pdf]
