# Supplementary material for: Assessing the allelotypic effect of two aminocyclopropane carboxylic acid synthase-encoding genes MdACS1 and MdACS3a on fruit ethylene production and softening in Malus
Source: Hortic Res. 2016 May 18;3:16024–. doi: 10.1038/hortres.2016.24 (PMC4870385; doi:10.1038/hortres.2016.24)
Supplement: Supplementary Table S2 [file hortres201624-s3.doc]

Table S2. Allele specific primers for genes *MdACS1* and *MdACS3a*

| Gene/Primer Name | Primer Sequence (5’ to 3’) |
| --- | --- |
| *MdACS1*/ACS1-5F | AGAGAGATGCCATTTTTGTTCGTAC |
| *MdACS1*/ACS1-5R | CCTACAAACTTGCGTGGGGATTATAAGTGT |
| *MdACS3a*/ACS3a-289F | CTTCCAGATTACTCCTCAAGCTTTA |
| *MdACS3b** | GTTCCAGATTACTCCAGAAGCGTTA |
| *MdACS3c** | TTTCCAAATCACTCCTCAAGCTTTG |
| *MdACS3a*/ACS3a-289R | AGTCTCTTTCTATTTGTCTTTATGTAGTTTC |
| *MdACS3b** | AGTCTCTCTCTGTTTGTATTTATGTAATTTT |
| *MdACS3c** | AGTCTCTCTCTATTTGTCTTTATGTAATTTT |

*The DNA bases of *MdACS3b* and *MdACS3c* highlighted in red show how they are discriminated from *MdACS3a* in the region covered by primers ACS3a-289F/R.
